# Creation of an algorithm for clinical decision support for treatment of opioid use disorder with buprenorphine in primary care

**DOI:** 10.1186/s13722-021-00222-0

**Published:** 2021-02-19

**Authors:** Adriane M. dela Cruz, Robrina Walker, Ronny Pipes, Sidarth Wakhlu, Madhukar H. Trivedi

**Affiliations:** Department of Psychiatry, University of Texas Southwestern Medical Center, 5323 Harry Hines Blvd, Dallas, TX 75390 USA

**Keywords:** Measurement based care, Buprenorphine, Clinical decision support

## Abstract

**Background:**

The treatment capacity for opioid use disorder (OUD) lags far behind the number of patients in need of treatment. Capacity is limited, in part, by the limited number of physicians who offer office based OUD treatment with buprenorphine. Measurement based care (MBC) has been proposed as a means to support primary care physicians in treating OUD. Here, we propose a set of measures and a clinical decision support algorithm to provide MBC for the treatment of OUD.

**Methods:**

We utilized literature search and expert consensus to identify measures for universal screening and symptom tracking. We used expert consensus to create the clinical decision support algorithm.

**Results:**

The Tobacco, Alcohol, Prescription medication, and other Substance use (TAPS) tool was selected as the best published measure for universal screening in primary care. No published measure was identified as appropriate for symptom tracking or medication adherence; therefore, we created the OUD Symptom Checklist from the DSM-5 criteria for OUD and the Patient Adherence Questionnaire for Opioid Use Disorder Treatment (PAQ-OUD) to assess medication adherence. We developed and present a clinical decision support algorithm to provide direct guidance regarding treatment interventions during the first 12 weeks of buprenorphine treatment.

**Conclusion:**

Creation of these tools is the necessary first step for implementation of MBC for the treatment of OUD with buprenorphine in primary care. Further work is needed to test the feasibility and acceptability of these tools.

*Trial Registration* ClinicalTrials.gov; NCT04059016; 16 August 2019; retrospectively registered; https://clinicaltrials.gov/ct2/show/NCT04059016

## Background

Opioid use disorder (OUD) has high rates of morbidity and mortality [1–3]. This disorder remains underdiagnosed and undertreated [4] despite the availability of FDA-approved medications with demonstrated efficacy in both specialty and primary care settings [5, 6]. The disconnect between the high efficacy of the medications and the limited number of patients receiving care may be related to the significant treatment implementation barriers faced by physicians, as evidenced by the limited number of physicians and mid-level providers able to prescribe buprenorphine for the treatment of OUD [7]. Different interventions to enhance physician knowledge and comfort with utilizing buprenorphine for the treatment of OUD in primary care have been developed [8], all with an ultimate goal to increase the number of patients receiving evidence-based care. Common elements among these models are the use of evidence-based pharmacotherapy, provision of education to providers, and integrating specialty and primary care.

Measurement based care (MBC), a systematic treatment approach in which disease symptoms, response to treatment, and medication adherence are used to inform clinical decisions [9], is one method for enhancing the number of primary care providers who can effectively diagnose and treat patients with OUD. The MBC approach promotes precision and consistency in disease assessment and treatment outcomes [10, 11] and has been suggested as an important element of treatment dissemination for OUD [9, 11]. Development and implementation of MBC has previously been effective for enhancing outcomes for patients with major depression, including enhancing rates of disease remission [12, 13]. The use of MBC for depression allows patients treated in primary care to achieve outcomes equivalent to patients receiving specialty care [14]. The critical elements of MBC are standardized assessments of symptoms and medication adherence, multi-step decision making for treatment, consistent follow up, and feedback to assist clinical decision making. While these critical elements can be implemented on paper, utilization of computer decision support software greatly enhances the feasibility of MBC [13] and allows for widespread scalability [15]. Our prior work has demonstrated the efficacy of this approach for successful treatment of patients with major depression in the primary care setting [16].

**Fig. 1:**
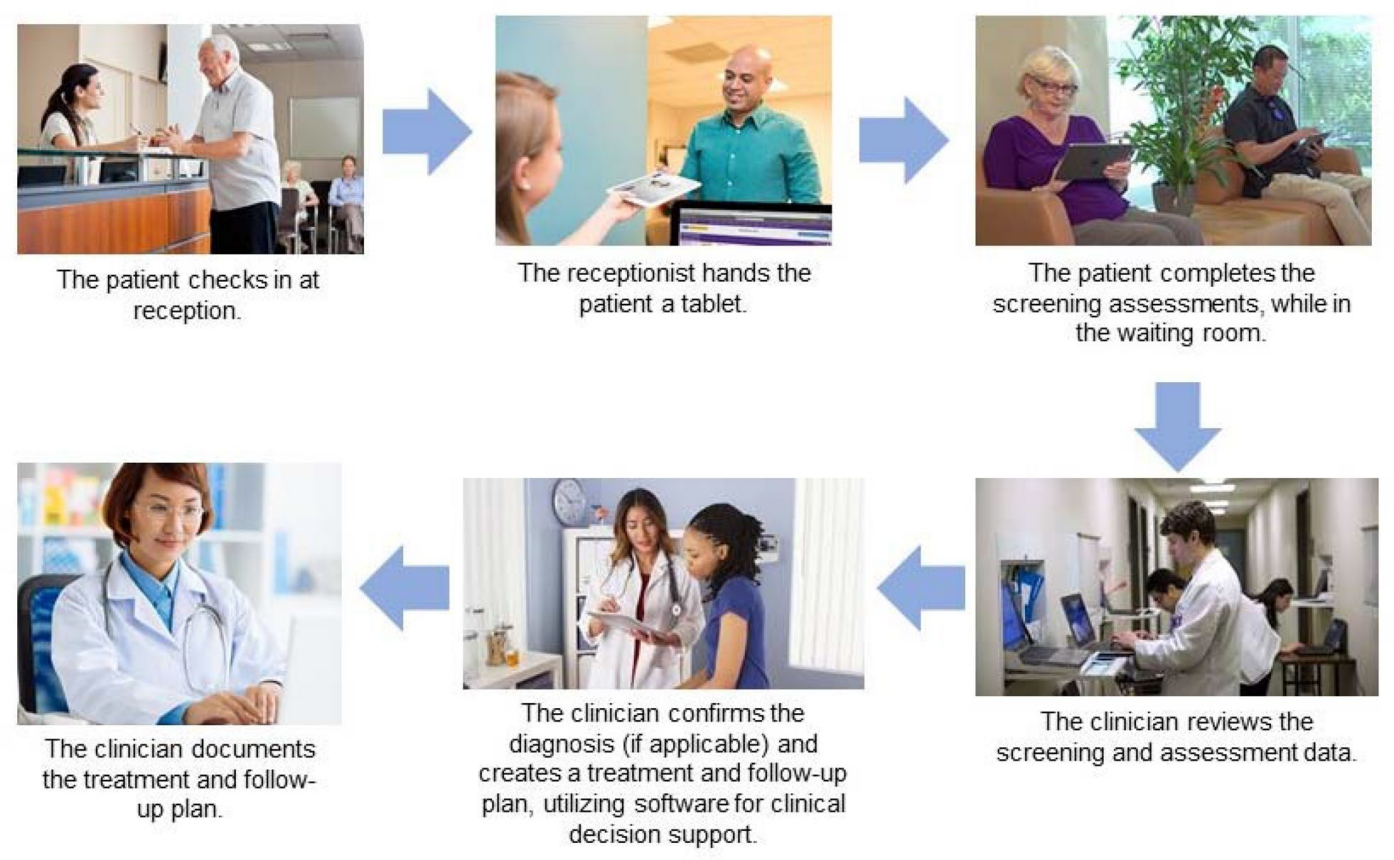
Typical Workflow for Measurement Based Care in Primary Care. In a typical measurement based care workflow, patients first complete a brief screening measure annually on a tablet computer in the clinic waiting room, prior to being seen by any clinical staff. Alternatively, the patient may complete the assessments after being seen by the medical assistant or nurse. Patients who screen positive on the brief measure then complete a more complete screening measure. The results of screening measures are reviewed by the clinician, who utilizes the screening results plus their clinical interview to make a diagnosis. The treating clinician utilizes the clinical decision support algorithm that is embedded in the software. At subsequent visits, patients for whom treatment has been initiated complete the medication adherence and symptom tracking questionnaires on the tablet computer

In a typical MBC workflow (Fig. 1) patients first complete a brief screening measure (e.g., PHQ-2). To meet the goal of universal screening, the brief screen is typically completed annually. Patients who screen positive on the brief measure then complete a more complete screening measure (e.g., PHQ-9). These measures are completed as self-reports to enhance report of symptoms and for clinic efficiency; most often, these measures are completed by the patient in the waiting room, either on paper or a tablet computer. The results of screening measures are thus immediately available to the treating clinician, who utilizes the screening results plus their clinical interview to make a diagnosis. The treating clinician may then choose to use a clinical decision support algorithm that is embedded in the software. The components of evidence based treatment and/or expert clinical consensus are converted into a set of “rules” to create the embedded algorithm that provides guidance regarding follow up time points and treatment recommendations. At all follow-up visits, the patient again completes a symptom tracking measure (e.g., PHQ-9) and a measure of medication adherence. These scores, the time since last assessment, and the treatment tactic used at the previous visit are automatically evaluated by the clinical decision support algorithm, which uses this information to provide further treatment recommendations. Thus, a single measure is utilized for screening and tracking treatment response, including determination of disease remission.

The National Drug Abuse Treatment Clinical Trials Network (CTN) funded the CTN-0090 MBC4OUD study as a one-year project to develop the essential components needed to implement MBC for OUD utilizing buprenorphine in the primary care setting. The MBC4OUD tools are built on the VitalSign6 (VS6) platform [17, 18], which utilizes health information technology tools and a web-based platform to enhance the quality of care for patients with depression in primary care. VitalSign6 employs a PCP-First model that emphasizes the role of primary care providers in screening for, diagnosing, and treating psychiatric illness in the primary care setting. In this report, we propose a set of measures and a clinical decision support algorithm to provide MBC for the office-based treatment of OUD with buprenorphine.

## Methods

Based on prior experience with MBC for depression, the following components were determined a priori to be necessary for creation of a clinical decision support tool: OUD screening measure, OUD symptom tracking measure, identification of critical decision time points for OUD treatment, assessment of medication adherence and side effects, treatment algorithm, and parameters for referral to specialty care. The treatment algorithm requires determining the recommended treatment course at each critical decision point based on symptom level.

### Screening and symptom tracking measures

Our goal was to design MBC4OUD with one assessment that could screen for OUD and could track OUD treatment response, similar to how MBC for depression is designed within VitalSign6. We hoped to identify a screening and symptom tracking measure for OUD that would serve the same role as the PHQ—a single measure for screening that could also be used to track treatment response.

The first step in identification of the needed measure was a literature review for published guidelines and recommendations for the treatment of opioid use disorder in both specialty and primary care settings. Resources reviewed included: Substance Abuse Mental Health Services Administration Treatment Improvement Protocol (SAMHSA TIP) 63: Medications for Opioid Use Disorder [19], The American Society for Addiction Medicine (ASAM) National Practice Guideline for the Use of Medications in the Treatment of Addiction Involving Opioid Use [20], SAMHSA Decisions in Recovery: Treatment for Opioid Use Disorder Handbook [21], Implementing Care for Alcohol & Other Drug Use in Medical Settings: An Extension of SBIRT [22], and Screening for Drug Use in General Medical Settings: Resource Guide [23].

These guidelines, plus a thorough search of the literature, were used to identity published measures that were candidates for (1) universal screening for OUD and (2) OUD symptom assessment and tracking. Given that the first step for successful implementation of MBC is universal screening, we sought an instrument that had been validated for use for universal screening in primary care settings with good sensitivity and specificity for the DSM diagnosis of OUD. We also sought an instrument that was brief and had been validated as a self-report.

### Clinical decision support algorithm

Creation of the clinical decision support algorithm required identifying three types of parameters: (1) time-points for patient assessment (i.e., critical decision time points), (2) categorization of level of treatment response, (3) assessment of medication adherence, and (4) types of recommended interventions. Together, these elements would allow the clinical decision support algorithm to provide a specific recommendation for treatment at a specific time based on the patient’s response to treatment assessed at the point of care. Practice guidelines cited above were used to identify recommendations for these parameters and criteria for determining poor response to treatment that necessitated referral to specialty care. Two authors (AMD, RW) used a series of discussions to draft the algorithm for clinical decision support. The content of the clinical decision support was then reviewed by an additional author (SW). The mechanics and logic were reviewed by an author (RP) who assisted with design and implementation of the VitalSign6 program; this author also generated the rules table utilized in creation of the MBC4OUD software program.

## Results

We identified six tools designed for screening for OUD and five for symptom tracking (Table 1).

**Table 1:** Instruments Considered for screening and symptom tracking for measurement based care

Measure	Screen or Outcome?	# of items	Time frame assessed	Self-report	References
AUDIT-C + 2	Screen	5	Past 3 months	Yes	Bradley et al. [22]
CRAFFT	Screen	9	Past year	Yes	Mitchell et al. [24]
NIDA Single Question	Screen	1	Past year	Yes	Smith et al. [25]
ASSIST-Lite	Primary & Secondary Screen	20^a^	Past 3 months	No	Ali et al. [26]
TAPS	Primary & Secondary Screen	4^b^	Past year^c^	Yes	McNeely et al. [27]
DSM Checklist	Secondary Screen & Outcome	11	Variable; traditionally past year	Possible	Marsden et al. [9]
Brief Addiction Monitor (BAM)	Outcome	17	Past 30 days	Possible	Cacciola et al. [28] Gaddy et al. [29]
Leeds Dependence Questionnaire (LDQ)	Outcome	10	Past week	Yes	Raistrick et al. [30] Heather et al. [31]
Treatment Outcomes Profile (TOP)	Outcome	20	Past 28 days	No	Marsden et al. [32]
Treatment Effectiveness Assessment (TEA)	Outcome	4	Not specified	Yes	Ling et al. [33]

^a^Primary screen is 7 items; additional items asked only if primary screen for that substance is endorsed

^b^Each item endorsed in TAPS1 leads to 2–3 follow up questions (TAPS2)

^c^Past 3 months in TAPS2

### Screening measure

We selected the Tobacco, Alcohol, Prescription medication, and other Substance use (TAPS) tool [27] as the screening measure. This measure utilized questions about past 12 and 3 month use of substances to categorize use as “no use,” “problem use,” or “higher risk” for each substance assessed. For this MBC4OUD project, any patient scoring > 0 for heroin or opioid is considered to need further assessment for OUD.

The TAPS tool was selected because the sensitivity and specificity for DSM-5 OUD have been assessed. The AUDIT-C + 2 [22] was considered but ultimately rejected because, while the sensitivity and specificity of this tool for detecting DSM alcohol use disorder has been assessed, the sensitivity and specificity for OUD has not been assessed. The CRAFFT [24] was considered but not selected as this tool has not been validated in adults. The NIDA Single Question [25] was not selected as this tool has been validated to asses for substance use disorders in general but not specifically for OUD. The ASSIST-Lite [26] was rejected as this tool has not validated as a self-report instrument. The authors considered modifying the NIDA Single Question to attempt to make it specific to OUD; however, the authors instead chose to use a tool with known sensitivity and specificity for detecting OUD in primary care settings. Thus, while the sensitivity of the TAPS tool for OUD (0.71–0.78) are potentially less than ideal and may miss a number of probable cases these properties have been characterized for an adult, outpatient, primary care population when used as a self report.

### Symptom tracking measure

It was determined, however, that the TAPS Tool would be insufficient to track symptoms over time for two reasons: (1) the TAPS assesses use over a 3-month period, while appropriate assessment of symptoms in patients with OUD occurs every 2–4 weeks [9] and (2) the TAPS Tool does not assess the full range of DSM symptoms. Two authors (AMD and RW) were unable to identify a published instrument for OUD that met these criteria and thus created the OUD Symptom Checklist (Appendix A). The OUD Symptom Checklist was derived from the DSM criteria (based on a proposal to utilize the DSM criteria in MBC for OUD [9]) and was created by altering the wording of each DSM criterion to a yes/no question regarding each symptom and by adjusting the words to reach an approximately eighth grade reading level (Flesh-Kincaid Grade Level 8.6). Each “yes” response equals one point, and points are summed for a total score. We added an additional question regarding use of any opioid other than buprenorphine in the past 2 weeks, as this is a clinically relevant feature for determining efficacy of response to buprenorphine treatment and disease remission.

### Clinical decision support algorithm

#### Time point for assessment

We set the time-point for re-assessment of patient symptoms at every 2 weeks, which continues until either a ‘full response’ to treatment is maintained for at least 4 weeks (after which the patient is re-evaluated every 4 weeks) or a maximum of 12 weeks elapsed with minimal treatment response (referral for specialty care would be provided). Clinicians may choose to see patients more frequently than once every 2 weeks, and the clinical decision support algorithm will consider the total time since initiating buprenorphine treatment to provide guidance. The former endpoint was based on SAMHSA guidelines for initiation of outpatient treatment of OUD with buprenorphine and expert consensus. The latter endpoint was based on the authors’ (AMD, RW, SW) expert consensus of a reasonable time period in which to attempt treatment in a primary care setting.

#### Categorization of treatment response

Through discussion, we identified two different potential heuristics for categorizing treatment response. Treatment response could be categorized as either current disease severity (i.e., no disease, mild-moderate, or severe) or as change from baseline (e.g., no/minimal treatment response, partial response, full response/remission). The advantage of utilizing current disease severity is that the approach emphasizes the goal of treating to disease remission. Treating to remission has been demonstrated to be critically important in depression [34] and is typically a treatment goal for acute illnesses. However, OUD treatment typically emphasizes harm reduction, improvement in symptoms, and decreases in use from baseline. SAMHSA and ASAM recognize that stopping *or reducing* the use of illicit opioids and the effects of illicit opioid use are appropriate treatment goals. Thus, to emphasize that improvement in symptoms is consistent with goals of care for OUD, we chose to categorize treatment response as change from baseline.

Thus, current symptom level is determined by the combination of total score on the OUD Symptom Checklist (i.e., number of DSM criteria) and the item assessing presence or absence of self-reported illicit opioid use in the past 2 weeks. Specifically, minimal or nonresponse is < 25% decrease from baseline total OUD symptom checklist score OR 25–75% decrease from baseline with use in past 2 weeks; partial response is 25–75% decrease from baseline OR > 75% decrease from baseline with use in past 2 weeks; and full response is > 75% decrease from baseline AND no use in past 2 weeks. We operationalized use in the past 2 weeks as a dichotomous variable (use or no use) rather than attempting to quantify changes in frequency or quantity of use from baseline. This choice was made in part for simplicity and ease of use in primary care. Thus, a patient with some opioid use, particularly intermittent use that is decreased from baseline, may still be considered to have a partial medication response. Additionally, we would not clinically consider a patient to have a full treatment response with any ongoing opioid use.

#### Assessment of adherence

Determination of medication adherence is a necessary first step for determining treatment response and is thus a critical step in MBC. The appropriate intervention with a patient who has not shown a treatment response and is not adherent to medication is to identify and address the reasons for non-adherence. In VitalSign6, medication adherence is assessed using the Patient Adherence Questionnaire (PAQ). The PAQ is a two item measure, with the first question assessing level of adherence (quantified as number of days in the past week in which medication was not taken) and item 2 assesses reasons for non-adherence. We modified the medication adherence questionnaire used in VitalSign6 to assess for medication adherence and named the new measure the Patient Adherence Questionnaire for Opioid Use Disorder Medication (PAQ-OUD; Appendix B). As in VitalSign6, we have defined taking medication 80% of the time (missing no more than 1 day in the past week) as adherent. In clinical practice, patients may be non-adherent to buprenorphine by either taking less than or more than prescribed, and each of these is queried in the first question on the measure. The second question assesses eleven different potential reasons for non-adherence (e.g., occurrence of side effects, inability to afford medications, ongoing cravings, experience of opioid withdrawal), and patients may endorse all that apply. If a patient endorses any form of medication non-adherence (e.g., taking more than prescribed or not taking medication on more than 1 day in the past week), the clinical decision support algorithm will alert the clinician to the non-adherence and advise the clinician to explore and address non-adherence prior to making any other changes. Specifically, clinicians are trained to ask the patient about the patient’s reasons for non-adherence, to work collaboratively with the patient to address the non-adherence, and to ask the patient about the patient’s perceived ability to address the barrier. Of note, when clinicians are trained to use MBC4OUD, they are instructed to consider increasing the dose of buprenorphine if patients report non-adherence to medication due to ongoing craving or opioid withdrawal.

#### Treatment interventions

**Fig. 2:**
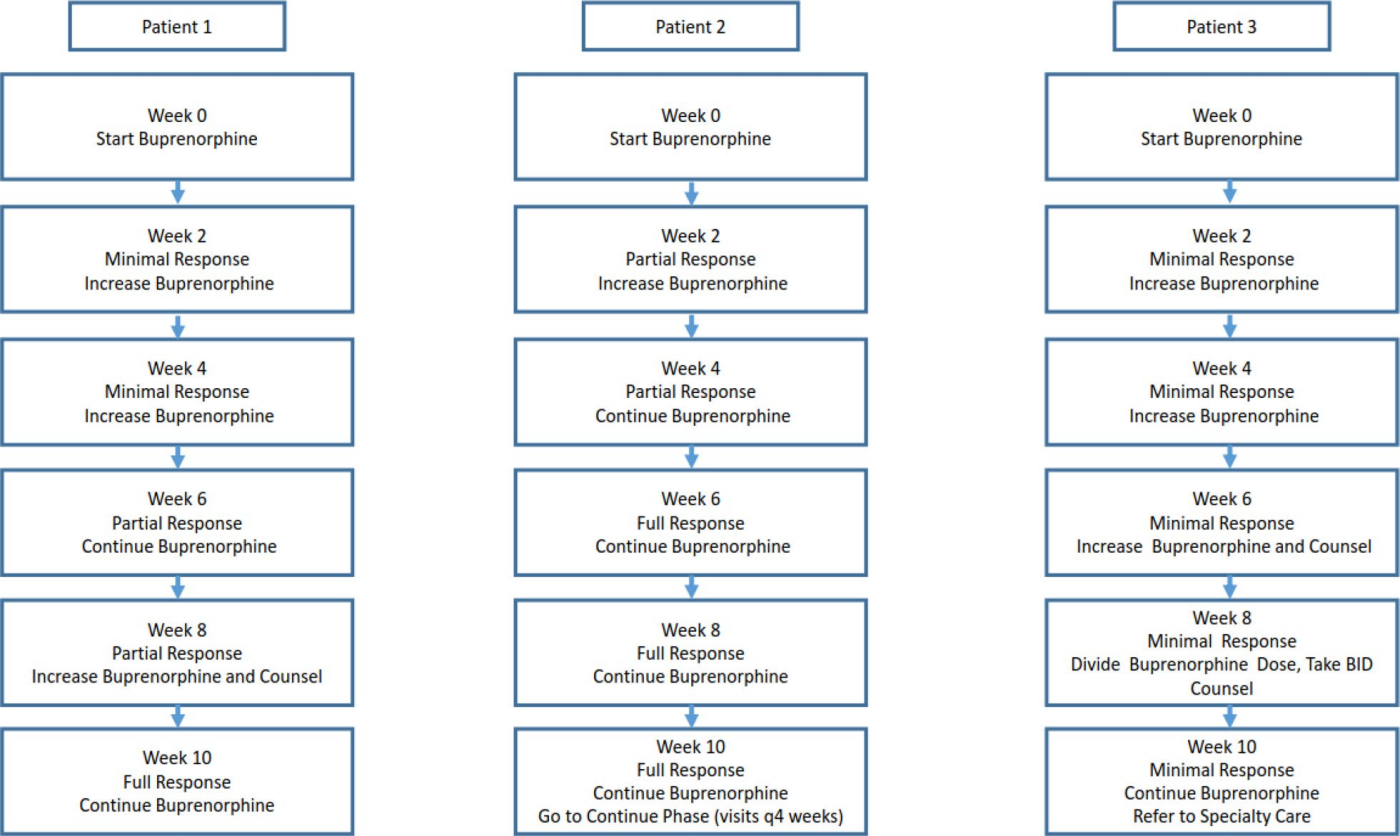
MBC4OUD Clinical Decision Support Schematic. This figure presents the treatment guidance for three hypothetical patients. Each box contains the time point of evaluation, treatment response shown by the patient at that time point, and the treatment guidance provided by the algorithm for the first 10 weeks of treatment for each patient. The typical situation in which the dose of buprenorphine would be continued unchanged in a patient with a partial response are those in which the patient is continuing to make improvements. A scenario that would follow the example of Patient 2 below is as follows: at Week 0, the patient is using 1 g of heroin daily and is initially stabilized on 8 mg buprenorphine daily. At the week 2 assessment, the patient report that heroin use has decreased in amount but is still occurring daily. In response, the buprenorphine dose is increased to 12 mg daily. At the next visit at week 4, the patient reports 2 days of a small amount of heroin use since last visit. The patient describes this use as “a waste of money” because he did not experience any of the desirable effects of heroin. The patient improvements related to DSM criteria (attending a family gathering, improved relationship with significant other, improvements in work attendance, etc.). The clinician chooses to maintain the current dose of buprenorphine at Week 4. At Week 6, the patient reports no use of heroin since the last visit and achieves full response

Identification of appropriate treatment interventions utilized in office based treatment of OUD with buprenorphine was made through review of treatment guidelines cited above as well as through expert consensus. The potential interventions identified for patients who are adherent to buprenorphine are: (1) no change, (2) increase dose of buprenorphine–naloxone not to exceed a recommended maximum total daily dose of buprenorphine 24 mg, (3) increase frequency of buprenorphine–naloxone dosing (i.e., twice daily dosing instead of once daily dosing), (4) add adjunctive counseling, and (5) refer to specialty care. In this context, “adjunctive counseling” refers to the series of interventions commonly described in the buprenorphine clinical trial literature as “medical management.” The interventions include routine assessment of ongoing drug use, validation and support of decreases in illicit drug use, and encouragement to explore community peer help groups [35]. The maximum dose of 24 mg was determined based on the FDA package label for Suboxone (buprenorphine–naloxone), which indicates no benefit above 24 mg/day. The treatment intervention of twice daily dosing (BID) was selected based on the clinical experience of the authors (AMD and SW) in caring for medically complex patients seen in specialty care. We have observed clinical benefit in a subset of outpatients, particularly those with a pain component. One author (AMD) created the grid indicating which interventions should be offered to which patients (based on treatment response) at which times, and this was reviewed by a second author (SW). Both authors are addiction psychiatrists with significant clinical experience in treating patients with OUD with buprenorphine and train others in this modality. Both authors agreed that the designed algorithm is aligned with treatment guidelines as well as expert clinical practice. The finalized treatment algorithm is presented in Table 2 and Fig. 2.

**Table 2:** Clinical decision support algorithm

Critical decision point	Critical status	Plan
Week 0 (CDP #1)	Symptomatic	Initiate buprenorphine treatment: adjust dose to lower end of therapeutic dose range
Week 2 (CDP #2)	Full response^2^	Continue current dose
	Partial response	Continue current dose OR Increase dose^3^
	Minimal or nonresponse	Increase dose
Week 4 (CDP #3)	Full response	Go to continuation phase if full response is sustained for at least 4 weeks. Otherwise, continue current does
	Partial response	Increase dose and counsel regarding behaviors to support recovery (including mutual help group attendance) OR Continue dose and counsel regarding behaviors to support recovery (including mutual help group attendance)
	Minimal or nonresponse	Increase dose and counsel regarding behaviors to support recovery (including mutual help group attendance) OR Continue dose and counsel regarding behaviors to support recovery (including mutual help group attendance)
Week 6 (CDP #4)	Full response	Go to continuation phase if full response is sustained for at least 4 weeks. Otherwise, continue current does
	Partial response	Increase dose and counsel regarding behaviors to support recovery (including mutual help group attendance) OR Divide daily dose in half, take medication BID and counsel regarding behaviors to support recovery (including mutual help group attendance)
	Minimal or nonresponse	Increase dose and counsel regarding behaviors to support recovery (including mutual help group attendance) OR Divide daily dose in half, take medication BID and counsel regarding behaviors to support recovery (including mutual help group attendance) OR Refer to specialty care. Continue current dose until patient is evaluated by specialist
Week 8 (CDP #5)^1^	Full response	Go to continuation phase if full response is sustained for at least 4 weeks. Otherwise, continue current does
	Partial response	Increase dose and counsel regarding behaviors to support recovery (including mutual help group attendance) OR Divide daily dose in half, take medication BID and counsel regarding behaviors to support recovery (including mutual help group attendance)
	Minimal or nonresponse	Divide daily dose in half, take medication BID and counsel regarding behaviors to support recovery (including mutual help group attendance) OR Refer to specialty care. Continue current dose until patient is evaluated by specialist
Week 10 (CDP #6)	Full response	Go to continuation phase if full response is sustained for at least 4 weeks. Otherwise, continue current does
	Partial response	Increase dose OR Counsel regarding behaviors to support recovery (including mutual help group attendance) OR Refer to specialty care. Continue current dose until patient is evaluated by specialist
	Minimal or nonresponse	Refer to specialty care. Continue current dose until patient is evaluated by specialist
Week 12 (CDP #7)	Full response	Go to continuation phase if full response is sustained for at least 4 weeks. Otherwise, continue current does
	Partial response	Increase dose OR Counsel regarding behaviors to support recovery (including mutual help group attendance) OR Refer to specialty care. Continue current dose until patient is evaluated by specialist
	Minimal or nonresponse	Refer to specialty care. Continue current dose until patient is evaluated by specialist

^1^For patients showing minimal or no response, total trial typically should not exceed 8 weeks.

^2^For patients with a partial response the trial may last up to 12 weeks to increase dose and implement counseling. Patients with only a partial response at any time point beyond 12 weeks should be considered for referral to specialty care. Minimal or nonresponse is < 25% decrease from baseline OR 25–75% decrease from baseline with use in past 2 weeks, partial response is 25–75% decrease from baseline OR > 75% decrease from baseline with use in past 2 weeks, and full response is > 75% decrease from baseline AND no use in past 2 weeks

^3^Per buprenorphine–naloxone SL package insert, dosages higher than 24 mg of buprenorphine-6 mg of naloxone have not demonstrated benefit. Maximum daily dose of buprenorphine not to exceed 24 mg

## Discussion

A major goal of CTN-0090 was creation of the tools (i.e., screening measure, symptom tracking measure, and clinical decision support algorithm) necessary to implement measurement based care for OUD with buprenorphine in primary care. We determined the TAPS Tool is an excellent measure to utilize for universal screening. We developed the OUD Symptom Checklist for baseline and follow-up assessment of response to treatment and the PAQ-OUD for assessment of medication adherence. We created a new treatment algorithm to provide clinical decision support. We have also created a software package for electronic implementation of these tools in routine clinical practice. The software package is built on the VitalSign6 platform, which has been utilized by clinics in our region since 2014 to provide measurement based care for depression. The next step in our work is testing these tools in primary care.

Potential strengths of our approach to implementing MBC for patients with OUD is that our method utilizes information gathered purposefully and directly from patients to evaluate and respond to OUD. This approach differs from other approaches that utilize information gathered from the electronic health record to identify patients at elevated risk for OUD. It is possible that our approach could be combined with other approaches; for example, patients identified by EMR tools as high risk for OUD could then be screened, assessed, and treated using our approach, which would allow for a targeted screening approach. A second strength is our choice of a screening instrument that has been well validated in primary care settings to allow for as efficient universal screening as possible. A third strength is that our approach can be implemented in typical practice without significant change to the clinic workflow (Fig. 1). Patients complete these self-report instruments in the clinic prior to seeing the physician or advanced practice provider; thus, the medical clinician has a complete report of the patient’s symptoms at the point-of-care. A fourth strength of our approach is a novel, robust clinical decision support algorithm that provides specific guidance to the medical clinician based on the patient’s change in symptoms from baseline and time in treatment. The tools have been combined in a software package that can be integrated with, or operate outside of, an electronic health record (EHR). A final strength is that our software is built on the VitalSign6 app software that has been utilized by primary care clinicians in a variety of settings for more than 5 years. Given these strengths, we propose that our MBC4OUD approach may be a useful and acceptable tool for delivering care for patients with OUD in primary care.

There are important limitations to our approach to MBC for OUD. Our approach is its limited focus on office based treatment of OUD with buprenorphine and is thus only applicable to clinicians who prescribe buprenorphine. We have not included support for buprenorphine induction in order to create a focused program and in recognition of the external resources for guidance in buprenorphine induction that already exist [e.g., information provided in waiver training, the FDA package label, supports provided through Provider Clinical Support System (PCSS-MAT)]. Our program does not assess comorbid conditions like chronic pain or psychiatric illness. The MBC4OUD and VitalSign6 depression modules can run concurrently to provide screening and treatment for co-occurring OUD and major depression. VitalSign6 includes optional measures to screen for major psychiatric illnesses. An additional limitation of the presented work is that the combination of screening and symptom tracking measures and clinical decision support algorithm have not yet been tested in clinical practice. A related limitation is that the OUD Symptom Checklist and PAQ-OUD, including our proposal that utilizing a modified version of DSM criteria is an appropriate strategy for assessing response to treatment, have not been validated. Additional work in validating the OUD Symptom Checklist, PAQ-OUD, and the algorithm is needed. Field trials will need to examine the question of whether item 10 on the OUD Symptom Checklist, which assess the effect to which patients feel the effects of opioids, should be reverse scored for patients on buprenorphine, as it is expected that buprenorphine treatment will block the effects of other opioids. Additionally, while we thoughtfully proposed the categorization of no, partial, and full treatment response, we acknowledge that these categories are arbitrary and have not been validated. Because of our decision to dichotomize use since last visit as present or absent, the system does not distinguish patients with decreasing or sporadic use in response to buprenorphine treatment from those with unchanged illicit use. Our decision to take a universal screening approach, as opposed to a targeted screening approach, may be regarded as a limitation. Specifically, there is concern that clinicians may abandon screening all together if they do not find the tools helpful for identifying patients in need of care, particularly as there are not currently financial incentives to support the time cost of screening for OUD. Targeted screening is often preferred due to the perception of enhanced efficiency and feasibility and concern for patient and clinician burden However, targeted screening approaches would require strong knowledge of risk factors for OUD, some of which may be poorly captured in the EHR. We believe the strengths of universal screening, which include de-stigmatizing OUD by making screening routine, outweigh the strength of a targeted approach. Of note, our approach does not explicitly require urine drug screen testing. Urine drug screen testing can confirm adherence to buprenorphine and that the patient has not used the opioids that are tested for in the last 1–3 days (time frame depends on substance pharmacokinetics). These tests are limited in that no urine drug screen can provide information on use over a longer time frame, and patients may use illicit or abused substances not assessed by the test. Given these cautions, we have not included a requirement for urine drug screen testing in determining the patient’s response to treatment, though these tests may be helpful for identifying recent illicit drug use and confirming buprenorphine adherence.

## Conclusion

Many models have been proposed for expanding access to care for patients with OUD; all of these models recognize the importance of medical clinicians outside of specialty addiction practice as critical to meeting the needs of patients with OUD [8]. Here, we propose a package for MBC for OUD that allows for a PCP-First approach [17] that has demonstrated efficacy in the treatment of depression and is a novel approach to the diagnosis and treatment of OUD. The work discussed here is the preliminary development of an approach that has not yet been tested and is thus limited. We believe this approach is an important step in addressing the gaps in care for patients with OUD.

## Data Availability

Data sharing is not applicable for this article as no datasets were generated or analyzed during the current study.

## Appendix A: Opioid Use Disorder (OUD) Symptom Checklist

The questions below are related to your opioid use. Opioids are a class of medicines used for the treatment of pain. They are also called narcotics. Some examples of opioids are hydrocodone, oxycodone, morphine, Fentanyl, and Dilaudid. Heroin is also an opioid.

In the past 2 weeks, have you:

1. Had times when you ended up using opioids more, or for longer, than you intended?
2. Wanted to cut down or stop using opioids, or tried to, but couldn’t? And have you wanted this or tried to do this more than once?
3. Spent a lot of time getting opioids, using opioids, or getting over the effects?
4. Experienced craving—a strong desire, or urge, to use opioids?
5. Found that using opioids—or being sick from using opioids—often interfered with the things you needed to do? For example, interfered with taking care of your home or family, caused job troubles, or school problems.
6. Continued to use opioids even though it was causing trouble with your family or friends?
7. Given up or cut back on activities that were important, interesting, or gave you pleasure, in order to use opioids? For example, you gave up or cut back on time with friends, work, or hobbies because of opioids?
8. Gotten into situations while using or after using opioids that increased your chances of getting hurt? For example, driving, swimming, using machinery, walking in a dangerous area, or having unsafe sex? And have you done this more than once?
9. Continued to use opioids even though it was making you feel sick, mentally or physically? For example, you continued to use opioids even though it was making you feel more depressed or anxious? Or adding to another health problem? Or after having had a memory blackout?
10. Had to use opioids much more than you once did to get the effect you want? OR found that the amount you usually use had much less effect than before?
11. Found that when the effects of opioids were wearing off, you had withdrawal symptoms? (Symptoms like trouble sleeping, irritability, anxiety, depression, being restless, muscle aches, diarrhea, fever, nausea, sweating, or feeling like things were not right?) OR have you used opioids to prevent or stop withdrawal symptoms?
  (At follow-up only, a twelfth item is presented):
12. Have you used any opioid, other than prescribed buprenorphine, in the past 2 weeks?

## Appendix B: Patient Adherence Questionnaire for Opioid Use Disorder Medication (PAQ-OUD)

1. How have you taken your prescribed buprenorphine medication during the last week? Please check the description that best describes your medication use.
  - ${\square}$ a. I have taken more medicine than prescribed on 1 or more days.
  - ${\square}$ b. I have taken my prescribed dose of medication every day without missing a day.
  - ${\square}$ c. I have missed taking my medication 1 day.
  - ${\square}$ d. I have missed taking my medication 2 or more days.
  - ${\square}$ e. I have taken less medicine than prescribed on 2 or more days.
  - ${\square}$ f. I have stopped taking my medication.
2. Please check all that apply for the past week.
  - ${\square}$ a. I have reduced the amount or days of my medication because I am feeling better.
  - ${\square}$ b. I have reduced the amount or days of my medication because of its side-effects.
  - ${\square}$ c. I have reduced the amount or days of my medication because I/my family cannot afford it.
  - ${\square}$ d. I have reduced the amount or days of my medication because I don’t believe I need it.
  - ${\square}$ e. I have not regularly taken my medication because I have trouble remembering to take it.
  - ${\square}$ f. I have not regularly taken my medication because the instructions are confusing or difficult to follow.
  - ${\square}$ g. I have not regularly taken my medication because it might cause other people to judge me.
  - ${\square}$ h. I have not regularly taken my medication because of the changes my doctor recommended I follow while on it (e.g., not taking other opioids, not taking illicit drugs).
  - ${\square}$ i. I have increased the amount of my medication because my pain is not controlled.
  - ${\square}$ j. I have increased the amount of my medication because I am having opioid cravings.
  - ${\square}$ k. I have increased the amount of my medication because I am experiencing symptoms of opioid withdrawal.

## Abbreviations

ASAM: American Society of Addiction Medicine
CDS: Clinical decision support
CTN: The National Drug Abuse Treatment Clinical Trials Network
EHR: Electronic Health Record
MBC: Measurement Based Care
OUD: Opioid Use Disorder
SAMHSA: Substance Abuse Mental Health Services Administration
TAPS: Tobacco, Alcohol, Prescription medication, and other Substance use
VS6: VitalSign6
